# Acute skin toxicity associated with a 1-week schedule of whole breast radiotherapy compared with a standard 3-week regimen delivered in the UK FAST-Forward Trial

**DOI:** 10.1016/j.radonc.2016.02.027

**Published:** 2016-07

**Authors:** A. Murray Brunt, Duncan Wheatley, John Yarnold, Navita Somaiah, Stephen Kelly, Adrian Harnett, Charlotte Coles, Andrew Goodman, Amit Bahl, Mark Churn, Rada Zotova, Mark Sydenham, Clare L Griffin, James P Morden, Judith M Bliss, on behalf of the FAST-Forward Trial Management Group

**Affiliations:** a University Hospitals of North Midlands & Keele University, Stoke-on-Trent, UK; b Royal Cornwall Hospital, UK; c The Institute of Cancer Research, London, UK; d The Institute of Cancer Research and The Royal Marsden, London, UK; e Derriford Hospital, Plymouth, UK; f Norfolk and Norwich University Hospitals NHS Foundation Trust, UK; g Cambridge University Hospital NHS Foundation Trust, UK; h Torbay District General Hospital, UK; i University Hospitals Bristol NHS Foundation Trust, UK; j Worcestershire Acute Hospitals NHS Trust, UK; k Mount Vernon Hospital, UK; l ICR-CTSU, The Institute of Cancer Research, London, UK

**Keywords:** Breast cancer, Radiotherapy, Hypofractionation

## Abstract

**Background and purpose:**

FAST-Forward is a phase 3 clinical trial testing a 1-week course of whole breast radiotherapy against the UK standard 3-week regimen after primary surgery for early breast cancer. Two acute skin toxicity substudies were undertaken to test the safety of the test schedules with respect to early skin reactions.

**Material and methods:**

Patients were randomly allocated to 40 Gy/15 fractions (F)/3-weeks, 27 Gy/5F/1-week or 26 Gy/5F/1-week. Acute breast skin reactions were graded using RTOG (first substudy) and CTCAE criteria v4.03 (second substudy) weekly during treatment and for 4 weeks after treatment ended. Primary endpoint was the proportion of patients within each treatment group with grade ⩾3 toxicity (RTOG and CTCAE, respectively) at any time from the start of radiotherapy to 4 weeks after completion.

**Results:**

190 and 162 patients were recruited. In the first substudy, evaluable patients with grade 3 RTOG toxicity were: 40 Gy/15F 6/44 (13.6%); 27 Gy/5F 5/51 (9.8%); 26 Gy/5F 3/52 (5.8%). In the second substudy, evaluable patients with grade 3 CTCAE toxicity were: 40 Gy/15F 0/43; 27 Gy/5F 1/41 (2.4%); 26 Gy/5F 0/53.

**Conclusions:**

Acute breast skin reactions with two 1-week schedules of whole breast radiotherapy under test in FAST-Forward were mild.

FAST-Forward is a randomised, multi-centre, three-group, non-blind, phase 3 clinical trial addressing the hypothesis that a 1-week course of adjuvant whole breast radiotherapy is at least as effective and safe as the UK standard 3-week regimen after primary surgery for early breast cancer. The primary endpoint for the main trial is local recurrence; Acute and late radiotherapy related adverse events are important secondary endpoints. Acute skin reactions are less sensitive to fraction size than late reacting normal tissues, so the lower total doses under test in the trial are expected to reduce their severity and duration, despite the shorter overall treatment time. In order to confirm these relationships, two acute toxicity substudies were undertaken during 2011 and 2013 in patients entered into the main trial. The need for two cohorts related mainly to a suboptimal choice of scoring system for the first substudy (described in Results). The results of both first and second toxicity substudies are presented here.

## Materials and methods

### Participants and treatment

In the main study, patients were allocated in a 1:1:1 ratio to 40 Gy/15F/3-weeks, 27 Gy/5F/1-week or 26 Gy/5F/1-week. Randomisation used computer generated random permuted blocks stratified by radiotherapy centre and tumour risk group. Centres telephoned The Institute of Cancer Research Clinical Trials and Statistics Unit randomisation service to ascertain the allocated treatment. Inclusion and exclusion criteria to the first and second acute toxicity substudies were as defined by the FAST-Forward protocol (http://public.ukcrn.org.uk/Search/StudyDetail.aspx?StudyID=10896), including patients who had undergone either breast conservation surgery or mastectomy (both substudies), with or without axillary dissection and/or cytotoxic therapies, and excluding patients requiring radiotherapy to regional lymph nodes other than lower axilla in standard tangential fields to breast/chest wall. Patients requiring a radiotherapy tumour bed boost dose were eligible for the first substudy, but were excluded from the second substudy in order to restrict the comparisons to the whole-breast component of treatment. At centres participating in the substudies, consecutive eligible patients were invited to participate at the time of consent to the main trial.

All patients provided written informed consent. FAST-Forward was approved by the national South East Coast Kent Research Ethics Committee (11/LO/0958) and the local Research and Development offices of all participating centres. The trial was sponsored by The Institute of Cancer Research and undertaken in accordance with the principles of Good Clinical Practice. This study is registered as an International Standard Randomised Controlled Trial, number ISRCTN19906132.

All patients underwent 3D CT-based treatment planning, including outlining of breast/chest wall to define clinical target volume and planning target volume (PTV) encompassed by medial and lateral tangential beams using megavoltage X-rays. The treatment plan was prescribed to a clinically relevant reference point and was optimised with 3D dose compensation to ensure >95% PTV received 95%, <5% PTV received ⩾105%, <2% PTV received ⩾107% and a global Dmax <110% of prescribed dose. Dose constraints for organs at risk were also specified in the protocol. Beam energies were usually 6 MV, but a mixture of higher energies could be used for large patients. Centres applying post-mastectomy bolus to the skin had to specify this intention prior to randomisation, including whether it was applied to whole skin or scar, for whole treatment or part there-of and the intended thickness. Treatment verification included daily portal imaging in patients randomised to 5-fraction schedules and imaging on the first three fractions and then weekly for patients randomised to 40 Gy/15F.

### Assessments

Acute reactions of the skin of the treated breast were graded using RTOG criteria for the first substudy. Following discussions with both the FAST-Forward Independent Data Monitoring Committee and Trial Steering Committee, it was agreed that this may not be the most appropriate scoring system in this context due to the inclusion of oedema in the RTOG system, since the primary concern was to score moist desquamation. Therefore it was agreed that a second substudy would be undertaken using standard CTCAE criteria (v4.03) (Appendix 3). Toxicity assessments were made by a healthcare professional at each participating centre using predesigned forms, see Appendix 4. The assessments were scheduled to be carried out weekly during treatment and for 4 weeks following the end of radiotherapy. In the second substudy, if moist desquamation beyond skin folds or creases (CTCAE grade 3) were seen during this time, weekly assessments were to continue until the reaction resolved to CTCAE grade 1 or less. If any assessment was missed, the centre contacted the patient by telephone to assess and grade (by asking the patient to describe the skin appearance and supplementing this by direct questions) acute skin reactions using a missed assessment form, see Appendix 5.

### Statistical considerations

The primary endpoint for each of the substudies was the proportion of patients within each treatment group with grade ⩾3 toxicity (RTOG and CTCAE respectively) at any time from the start of radiotherapy to 4 weeks after completion of radiotherapy. The secondary endpoints were clinical assessment of (i) any acute skin toxicity, defined as the worst grade reported from the start of radiotherapy to 4 weeks post-radiotherapy, (ii) acute skin toxicity during radiotherapy, defined as the worst grade reported from the start to the end of radiotherapy, (iii) acute skin toxicity post radiotherapy, defined as the worst grade reported at completion of radiotherapy until at least 4 weeks post radiotherapy and (iv) adherence to the acute toxicity assessments

Principal analyses were based on the evaluable population, defined as all patients randomised into the study receiving at least one fraction of radiotherapy (regardless of whether they were later found to be ineligible or a protocol violator) and with complete data, or, at most, one missing toxicity assessment. For the first substudy which included some patients receiving a boost, only non-boost assessments were used to define inclusion in the evaluable population. The required number of assessments for a 40 Gy/15F patient was 7 (3 during and 4 post-radiotherapy) and 5 for 27 Gy/5F and 26 Gy/5F patients (1 during and 4 post-radiotherapy). Patients who switched to receive a different trial treatment after randomisation were analysed according to treatment received rather than randomised treatment. For the primary endpoint, the proportion of patients within each treatment group with grade ⩾3 RTOG toxicity (first substudy) or grade ⩾3 CTCAE (second substudy) toxicity was estimated with associated upper one-sided 95% confidence interval. Secondary endpoints were estimated as frequencies and percentages, with the prevalence of grades ⩾1, ⩾2 and ⩾3 toxicity at each time-point presented graphically. Adherence to toxicity assessments was estimated as frequencies and percentages. Toxicity data were included as reported, there were no restrictions based on the date of assessment. The acute toxicity substudy was designed to be non-comparative so no statistical comparisons were made across the treatment groups.

Each substudy aimed to recruit approximately 150 evaluable patients (50 per group) in order to exclude a within-group rate of grade ⩾3 acute skin reactions of over 11% compared to target rate of under 3% (89% power and one-sided 7.9% significance level).

## Results

Recruitment to the first acute toxicity substudy opened in November 2011 and closed in May 2012. Of 194 potentially eligible patients recruited in substudy centres, 190 patients consented (63, 63 and 64 in the 40 Gy/15F, 27 Gy/5F and 26 Gy/5F groups, respectively). Recruitment was performed at 9 treating centres across the UK with the infrastructure to carry out weekly toxicity assessments.

Recruitment to the second acute toxicity substudy opened in April 2013 and closed in February 2014. Of 269 potentially eligible patients recruited in the 8 substudy centres, 162 patients consented (55, 44 and 63 in the 40 Gy/15F, 27 Gy/5F and 26 Gy/5F groups, respectively). Two patients subsequently withdrew consent for all data to be used. The number of patients potentially eligible, consented, receiving allocated radiotherapy and evaluable for the primary endpoint for each substudy is given in Appendix 1.

Baseline characteristics are presented for all consenting patients recruited to the substudy, and were broadly comparable with those of the whole trial populations (Appendix 2). Imbalances in numbers allocated to different groups are within expectation of random variation. Treatment characteristics and comorbidity data were comparable to those collected from all patients in the main trial, and there were no obvious imbalances in features between toxicity study groups (data not shown).

Radiotherapy treatment characteristics (for each substudy separately) are presented in Table 1, suggesting no significant imbalances between groups. Compliance to toxicity assessments by treatment group is shown in Table 2. For the first acute toxicity substudy, worst RTOG grade experienced by treatment received is reported in Table 3a. The proportions of evaluable patients with grade 3 RTOG toxicity during the acute phase were as follows: 40 Gy/15F 6/44 (13.6%); 27 Gy/5F 5/51 (9.8%); 26 Gy/5F 3/52 (5.8%). Twenty-nine patients in the first substudy received a boost to their treatment, of which 22 were evaluable. No evidence of a higher rate of grade 3 toxicity was observed in this subset of patients (40 Gy/15F 1/9 (11.1%); 27 Gy/5F 0/10; 26 Gy/5F 0/3).

**Fig. 1:**
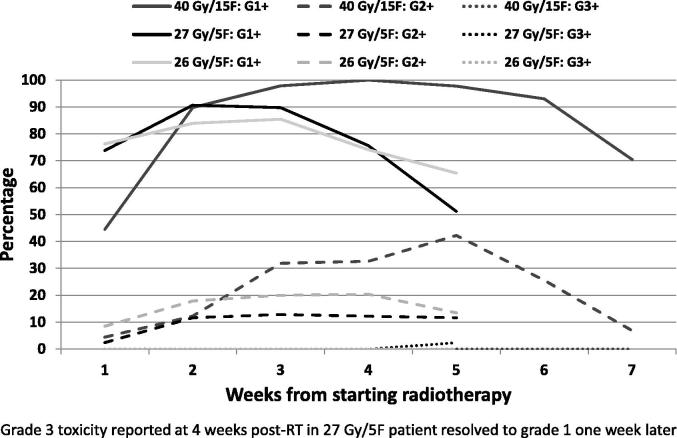
Acute toxicity substudy 2 – Prevalence of grade 1+, grade 2+ and grade 3+ CTCAE toxicity.

For the second acute toxicity substudy, worst CTCAE grade experienced by treatment received is reported in Table 3b. The proportions of evaluable patients with a grade 3 CTCAE toxicity during the acute phase were as follows: 40 Gy/15F 0/43; 27 Gy/5F 1/41 (2.4%); 26 Gy/5F 0/53. Grade 2 toxicity was largely due to moderate to brisk erythema, with only 3 patients with moderate oedema (two 40 Gy/15F and one 26 Gy/5F patient), see Table 3c. CTCAE toxicity score reported at each time point is presented graphically during radiotherapy and post radiotherapy, see Fig. 1. A single patient in the second cohort randomised to the 27 Gy/5F group after mastectomy, adjuvant cytotoxic chemotherapy and chest wall radiotherapy with bolus was assessed as a CTCAE grade 3 toxicity recorded after a missed assessment by telephone appointment 36 days after start of treatment, having been assessed as grade 2 seven days before and assessed with grade 1 erythema seven days later.

## Discussion

The acute skin toxicity substudies were not designed to involve statistical hypothesis testing across treatment groups, but to confirm low incidence rates of clinically significant acute skin toxicity associated with each schedule. On this basis, the results raise no concerns that the 5-day schedules lead to more severe or longer-lasting acute skin reactions compared to 40 Gy in 15 fractions. The prevalence rates summarised in Fig. 1 suggest that erythema after the 1-week schedule is less intense and settles about 2 weeks earlier than after the 3-week schedule. An imbalance in numbers in the 27 Gy group is attributed to the play of chance, since consent to participate in the substudy was obtained prior to allocation of randomised treatment. Overall compliance with the toxicity assessments was high, making it unlikely that patients developing severe skin reactions were under-reported. No suggestion of a dose response is noted between the 2 dose levels of the 5-fraction test regimens. The results of the first toxicity substudy are consistent with those from the second substudy, despite the unhelpful inclusion of oedema with erythema and desquamation in the RTOG system used for scoring early skin reactions.

The mildness of the acute skin toxicity associated with the 5-fraction regimens was expected. A series of classic studies investigating the dependence of acute skin reactions on total dose, fraction size, inter-fraction interval and overall treatment time was undertaken in the 1980 s and 1990 s by Turesson and colleagues, using reflectance spectrophotometry to quantify erythema and clinical grading to score moist desquamation [1], [2], [3]. These confirmed the absence of a treatment time effect for the first 4 weeks of radiotherapy delivered using fraction sizes of 2.0 Gy and 4.0 Gy delivered using 12 MeV electrons to the internal mammary chain daily or twice per week [1]. Using the same experimental system, whereby different fractionation regimens were delivered to right and left internal mammary chains of the same patient, weak dependence of erythema and desquamation on fraction size was demonstrated, with estimates of *α*/*β* ratios between 7.5 and 11.2 Gy [2]. In the context of the FAST-Forward trial, the reductions in total dose, from 40 Gy to 27 Gy and 26 Gy appear to compensate for increased dose per fraction where acute skin reactions are concerned. In conclusion, the acute skin toxicity substudies conducted in patients entered into the FAST-Forward trial raise no concerns, while long-term outcomes on the total trial population of 4000 patients are awaited.

## Role of the funding source

The FAST-Forward trial is funded by the National Institute for Health Research – Health Technology Assessment programme (reference 09/01/47). The views and opinions expressed therein are those of the authors and do not necessarily reflect those of the Health Technology Assessment programme, NIHR, NHS or the Department of Health. Cancer Research UK provide core funding to ICR-CTSU (Grant C1491/A9895). Neither funder were involved in the study design, in the collection, analysis and interpretation of data; in the writing of the manuscript; and in the decision to submit the manuscript for publication. C.C. is supported by the Cambridge National Institute of Health Research Biomedical Research Centre.

## Ethical considerations

Release of data from the FAST-Forward acute toxicity substudies was approved by both the Independent Data Monitoring Committee and Trial Steering Committee. All trial participants provided written informed consent.

## Conflict of interest statement

No authors have conflicts of interest to declare

## Figures and Tables

**Table 1:** Radiotherapy details of patients in first and second acute toxicity substudies.

Radiotherapy (RT) details	Acute toxicity substudy 1			Acute toxicity substudy 2		
40 Gy/15F	27 Gy/5F	26 Gy/5F	40 Gy/15F	27 Gy/5F	26 Gy/5F
*N* = 64^a^	*N* = 62	*N* = 63	*N* = 55^b^	*N* = 44	*N* = 61
*N* (%)	*N* (%)	*N* (%)	*N* (%)	*N* (%)	*N* (%)
*Radiotherapy received*						
Yes	64 (100)	62 (100)	63 (100)	54 (98)	44 (100)	61 (100)
No	0	1 (2)^c^	0	1 (2)^d^	0	0

*Boost received*						
Yes	11 (17)	11 (18)	7 (11)	0	0	0
No	53 (83)	51 (82)	56 (89)	54 (100)	44 (100)	61 (100)

*Treatment duration in patients receiving no boost (including weekends and bank holidays)*^e^						
Median (IQR)	22 (21–23)	5 (5–7)	7 (5–7)	21 (20–22)	6 (5–7)	6 (5–7)
Range	1–25	5–11	1–9	15–23	5-8	5-8

*Treatment duration in patients receiving boost (including weekends and bank holidays)*						
Median (IQR)	30 (26–33)	18 (12–20)	14 (12–17)	–	–	–
Range	22–35	7–20	7–21	–	–	–

*Whole breast – total fractions*						
5	0	62 (100)	63 (100)	0	44 (100)	61 (100)
15	63 (98)	0	0	54 (100)	0	0
Other^f^	1 (2)	0	0	0	0	0

*Whole breast – total dose (Gy)*						
26	0	0	63 (100)	0	0	61 (100)
27	0	62 (100)	0	0	44 (100)	0
40	63 (98)	0	0	54 (100)	0	0
Other^6^	1 (2)	0	0	0	0	0

*Bolus used (mastectomy patients only)*						
Yes	1 (100)	1 (33)	3 (50)	2 (40)	1 (50)	5 (100)
No	0	2 (67)	3 (50)	3 (60)	1 (50)	0

*Dose homogeneity constraints achieved*						
Yes	62 (97)	61 (98)	60 (95)	53 (98)	44 (100)	60 (98)
No	2 (3)	1 (2)	3 (5)	1 (2)	0	1 (2)

*Organs at risk dose constraints achieved*						
Yes	64 (100)	61 (98)	62 (98)	53 (98)	44 (100)	61 (100)
No	0	1 (2)	1 (2)	1 (2)	0	0

*Any deviations from RT*						
Yes	1 (2)	0	0	1 (2)	0	0
No	63 (98)	62 (100)	63 (100)	53 (98)	44 (100)	61 (100)

*Whole breast RT extended by >3 days*						
Yes	0	0	0	0	0	0
No	64 (100)	62 (100)	63 (100)	54 (100)	44 (100)	61 (100)

a Includes one patient randomised to receive 26 Gy/5F who was treated with 40 Gy/15F due to patient choice, this patient is included in 40 Gy/15F group for all subsequent analyses.

b Includes one patient randomised to receive 26 Gy/5F who was treated with 40 Gy/15F due to coverage issues, this patient is included in 40 Gy/15F group for all subsequent analyses.

c Patient refused any radiotherapy after randomisation.

d Patient withdrew consent prior to radiotherapy.

e Treatment duration for 40 Gy/15F patients with no boost is 19 days if it starts on a Monday and 21 days if it starts Tuesday to Friday. 27 Gy/5F and 26 Gy/5F patients have 5 days treatment if it starts on a Monday and 7 days if it starts Tuesday to Friday.

f One patient randomised to 40 Gy/15F who chose to discontinue radiotherapy after receiving 32 Gy/12F.

**Table 2:** Overall compliance to toxicity assessments during and post radiotherapy.

Total number of acute toxicity assessments during and post treatment	Acute toxicity substudy 1			Acute toxicity substudy 2		
40 Gy/15F	27 Gy/5F	26 Gy/5F	40 Gy/15F	27 Gy/5F	26 Gy/5F
*N* = 64	*N* = 62	*N* = 63	*N* = 55	*N* = 44	*N* = 61
*N* (%)	*N* (%)	*N* (%)	*N* (%)	*N* (%)	*N* (%)
Expected number of assessments	7	5	5	7	5	5

*Number of completed assessments*^a^						
0	2 (3)	0	1 (2)	6 (11)	0	1 (2)
1	3 (5)	3 (5)	4 (6)	0	0	1 (2)
2	2 (3)	3 (5)	0	0	1 (2)	1 (2)
3	5 (8)	5 (8)	6 (10)	1 (2)	2 (5)	5 (8)
4	1 (2)	16 (26)	14 (22)	1 (2)	5 (11)	7 (11)
5	7 (11)	35 (56)	38 (60)	4 (7)	36 (82)	46 (75)
6	14 (22)	–	–	9 (16)	–	–
7^b^	30 (47)	–	–	34 (62)	–	–
Total number evaluable	44	51	52	43	41	53

a Only includes non-boost assessments (First substudy).

b Includes one patient with 4 on-treatment assessments +4 post-treatment assessments.

**Table 3a:** Acute skin toxicities reported. Acute toxicity substudy 1 – Worst acute CTCAE score according to treatment.

Worst RTOG grade (on or post RT)	40 Gy/15F	27 Gy/5F	26 Gy/5F
*N* = 44	*N* = 51	*N* = 52
*N* (%)^a^	*N* (%)^a^	*N* (%)^a^
0	0	2 (4)	3 (6)
1	14 (32)	24 (47)	32 (62)
2	24 (55)	20 (39)	14 (27)
3	6 (14)	5 (10)	3 (6)
4	0	0	0
Percentage of RTOG grade 3+ (upper limit of one-sided 95% CI)	13.6 (25.2)%	9.8 (19.5%)	5.8 (14.2)%

a Percentages calculated from those evaluable.

**Table 3b:** Acute toxicity substudy 2 – Worst acute CTCAE score according to treatment.

CTCAE grade	40 Gy/15F	27 Gy/5F	26 Gy/5F
*N* = 43	*N* = 41	*N* = 53
*N* (%)^a^	*N* (%)^a^	*N* (%)^a^
0	0	3 (7)	3 (6)
1	21 (49)	26 (63)	31 (58)
2	22 (51)	11 (27)	19 (36)
3	0	1 (2)^b^	0
4	0	0	0
Proportion grade 3+ (upper limit of one-sided 95% CI)	0 (6.7)%	2.4 (11.1)%	0 (5.5)%

a Percentages calculated from those evaluable.

b Grade 3 toxicity reported at 4 weeks post-RT resolved to grade 1 one week later.

**Table 3c:** Acute toxicity substudy 2 – Nature of grade 1 and 2 toxicities according to treatment.

CTCAE grade 1 and 2 symptoms	40 Gy/15F	27 Gy/5F	26 Gy/5F
*N* (%)^a^	*N* (%)^a^	*N* (%)^a^
*Grade 1*			
Faint erythema	42 (98)	38 (93)	47 (89)
Dry desquamation	7 (16)	1 (2)	8 (15)

*Grade 2*			
Moderate to brisk erythema	20 (47)	11 (27)	16 (30)
Patchy moist desquamation confined to skin folds/creases	8 (19)	2 (5)	6 (11)
Moderate oedema	2 (5)	0	1 (2)

a Percentages calculated from those evaluable.
