# Supplementary material for: Acute skin toxicity associated with a 1-week schedule of whole breast radiotherapy compared with a standard 3-week regimen delivered in the UK FAST-Forward Trial
Source: Radiother Oncol. 2016 Jul;120(1):114–8. doi: 10.1016/j.radonc.2016.02.027 (PMC4998960; doi:10.1016/j.radonc.2016.02.027)
Supplement: Supplementary appendix [file mmc1.pdf]

SUPPLEMENTARY FILES

Appendix 1: Flow diagram of participation in the first and second acute toxicity substudies

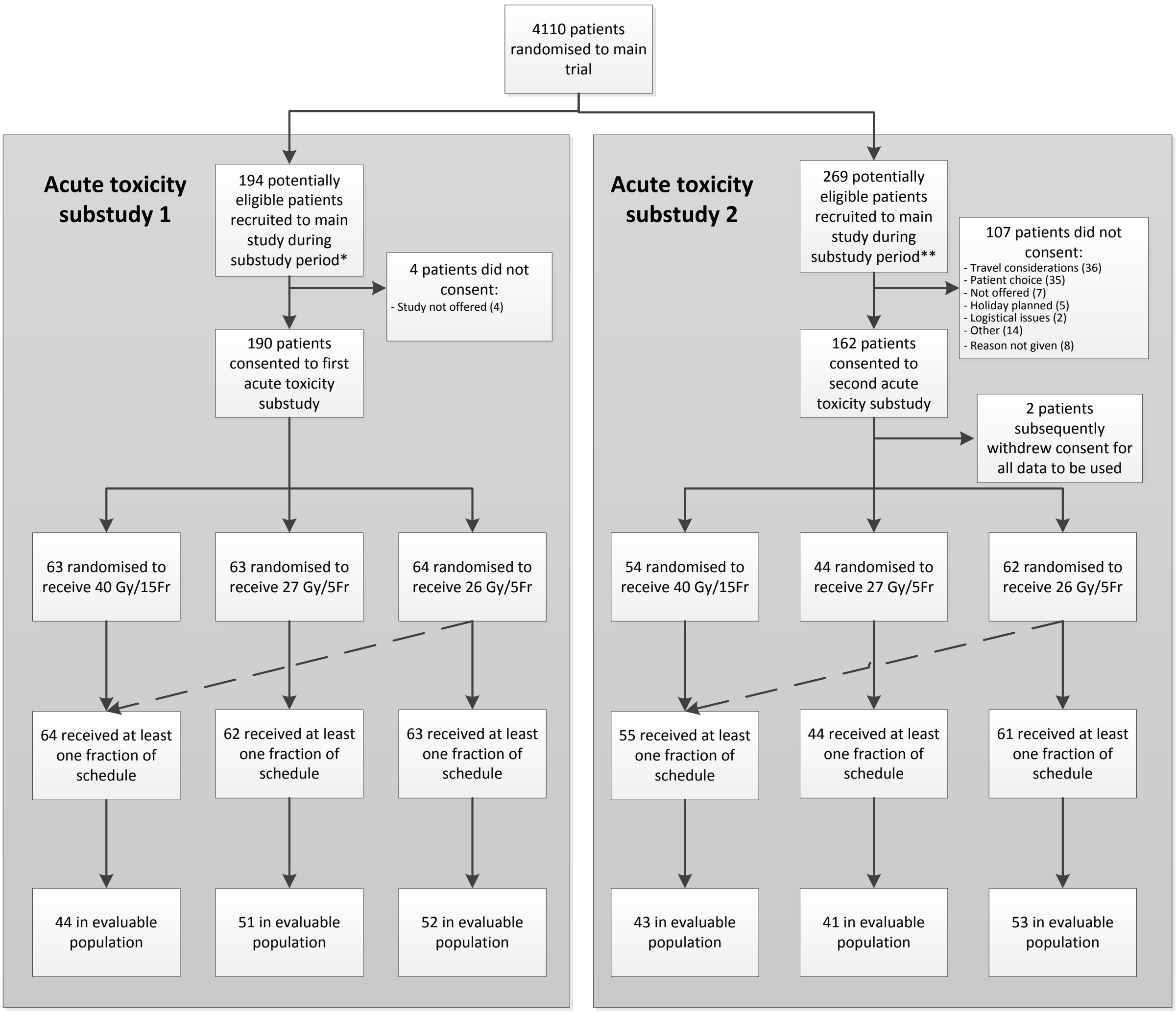

Appendix 2: Baseline characteristics of patients in first and second acute toxicity substudies

Baseline characteristics	Acute toxicity substudy 1			Acute toxicity substudy 2		
	40 Gy/15F N=63 N (%)	27 Gy/5F N=63 N (%)	26 Gy/5F N=64 N (%)	40Gy/15fr N=54 N (%)	27Gy/5fr N=44 N (%)	26Gy/5fr N=62 N (%)
**Age**						
Median (IQR)	63 (57 - 69)	65 (54 - 68)	63 (55 - 68)	60 (54-66)	61 (56-70)	61 (54-64)
Range	46-87	41-82	44-86	47-81	49-84	42-78
**Age category**						
<50	5 (8)	11 (17)	10 (16)	3 (5)	2 (5)	6 (10)
≥50	58 (92)	52 (83)	54 (84)	52 (95)	42 (95)	57 (90)
**Tumour grade**						
1	15 (24)	16 (25)	17 (27)	14 (25)	10 (23)	16 (25)
2	28 (44)	31 (49)	33 (52)	27 (49)	23 (52)	34 (54)
3	20 (32)	16 (25)	14 (22)	14 (25)	11 (25)	13 (21)
**Risk group**						
Low (≥50 & grade 1 or 2)	40 (63)	39 (62)	43 (67)	38 (69)	31 (70)	45 (71)
High (<50 and/or grade 3)	23 (37)	24 (38)	21 (33)	17 (31)	13 (30)	18 (29)
**Boost planned**						
No	54 (86)	52 (83)	59 (92)	55 (100)	44 (100)	63 (100)
Yes	9 (14)	11 (17)	5 (8)	0	0	0

Appendix 3: Modified RTOG and CTCAEv4.03 definitions used in first and second acute skin toxicity sub-studies

Acute toxicity substudy 1

***Modified RTOG**	**Description**
Grade 1	Faint/dull erythema
Grade 2	Tender/bright erythema +/- dry desquamation
Grade 3	Patchy moist desquamation, moderate oedema
Grade 4	Confluent moist desquamation, pitting oedema

Acute toxicity substudy 2

**CTCAEv4.03**	**Description**
Grade 1	Faint erythema or dry desquamation
Grade 2	Moderate to brisk erythema; patchy moist desquamation, mostly confined to skin fold; moderate oedema
Grade 3	Moist desquamation in areas other than skin folds and creases; bleeding induced by minor trauma or abrasion
Grade 4	Life threatening consequences; skin necrosis or ulceration of full thickness dermis; spontaneous bleeding from involved site; skin graft indicated

*The inclusion of oedema in the modified RTOG system proved unhelpful in this particular context, since the primary concern was to score moist desquamation; hence the second substudy was undertaken using CTCAE criteria

Appendix 4: Toxicity assessment form

FAST-Forward

Skin Toxicity During Radiotherapy: Control group

RT Centre ______ Trial number FF ______

Referring Hospital ______

Patient's Initials ______ Date of Birth ______ (Day Month Year)

SKIN TOXICITY ASSESSMENT DURING RADIOTHERAPY: CONTROL GROUP WITH NO BOOST

Date RT started ______ (Day Month Year)

Assessments are to be carried out by a healthcare professional weekly. Please specify date of each assessment and tick the appropriate box indicating grade according to the CTCAE scoring criteria. If grade 1 or 2 please also tick the relevant symptom(s)

	Before RT begins	During 1st week	During 2nd week	During 3rd week	Only complete if RT duration>3 weeks**	
**Date of assessment**						
**CTCAE grade**						
**0** (no toxicity)						
**1** *Please tick all relevant symptoms*						
faint erythema						
dry desquamation						
**2** *Please tick all relevant symptoms*						
moderate to brisk erythema						
patchy moist desquamation confined to skin folds/creases						
moderate oedema						
**3**						
**4**						
**5**						
Not assessed *						

**** If any assessment was not done please contact the patient and complete the Missed Assessment Form***

*** If radiotherapy is delayed and extends beyond 3 weeks then additional weekly toxicity assessments will be required during radiotherapy – please complete as required*

CTCAE v4.03 Dermatitis radiation	
1	Faint erythema or dry desquamation
2	Moderate to brisk erythema; patchy moist desquamation, mostly confined to skin folds and creases; moderate edema
3	Moist desquamation in areas other than skin folds and creases; bleeding induced by minor trauma or abrasion
4	Life-threatening consequences; skin necrosis or ulceration of full thickness dermis; spontaneous bleeding from involved site; skin graft indicated
5	Death

Please add any other relevant comments in the space provided

Signature ______

Print Name ______ Date form completed ______ (Day Month Year)

For Trials office use only

Received	Entered	Checked
TRAQS ☐ Initials	Initials	Initials

Appendix 5: Missed assessment form

FAST- Forward

Missed Assessment Form

RT Centre

Referring Hospital

Patient's Initials

Trial number FF

Date of Birth (Day Month Year)

TO BE COMPLETED IF A PATIENT DOES NOT ATTEND FOR A TOXICITY ASSESSMENT

Was the patient called if they failed to turn up for a toxicity assessment? Yes* ☐ No** ☐

** If yes, please complete the rest of this form*

*** If no, please specify reason*

Date of telephone assessment (Day Month Year)

The patient should be asked the following questions:

Please state reason for not attending for an assessment

Has there been any change in skin colour since last week?

Yes, less pink/red ☐ Yes, more pink/red ☐ No, same colour ☐

Is there been any evidence of skin weeping or oozing?

Yes* ☐ No ☐

** If yes, please specify the site* Skin creases/folds ☐ Other ☐

Signature

Print Name

Date form completed (Day Month Year)

Received

TRAQS ☐ Initials

Entered

Initials

Checked

Initials

For Trials office use only
